# Weary

**Published:** 1891-10

**Authors:** Amarala Martin

**Affiliations:** Cairo, Ill.


### WEARY.

For Hall's Journal of Health.

By Amarala Martin.

The dreary days, the dreary days,
Drag all their darksome lengths along,
They crush from life all happy song,
And leave me but its saddest lays.

For, oh! my loved of spirit-land,
Seem far away though oft so near—
I may not their dear voices hear,
Nor clasp again a gentle hand.

In the dim future, as the past,
While my whole life doth for them yearn,
I'll watch and wait for their return,
Faithful for aye, true to the last.

Oh, may I so live here, that there
In that bright world, I may not feel
Remorseful mem'ries o'er me steal,
From out the shadows of despair.

